# Supplementary material for: LinkML: an open data modeling framework
Source: Gigascience. 2025 Dec 12;15:giaf152. doi: 10.1093/gigascience/giaf152 (PMC12993438; doi:10.1093/gigascience/giaf152)
Supplement: Supplementary file 1 [file giaf152_authors_response_to_reviewer_comments_original_submission.pdf]

Author's Response To Reviewer Comments

Dear GigaScience Editors,

Thank you for your review of our manuscript, "LinkML: An Open Data Modeling Framework" (GIGA-D-25-00274). We have revised it to address the reviewers' concerns.

Reviewer #1 said:
The authors assert that the approach complies with FAIR data standards, the first principle of which is persistent identification. While persistent identification is mentioned in numerous places in the article we don't find any detail on why the identifiers used are persistent. Perhaps readers more conversant in the domains from which the authors seem to come would understand this but we do not. We suggest a separate section or at least some detailed footnotes or citations to detail the way in which persistent identification fits within the LinkML framework.

This is a great point. We have now added a section explaining why the identifiers are persistent.

Reviewer #2 said:
it seems to me that there is a critical section missing that positions the framework in the existing ecosystem, not only with regard to formats, but especially with regard to other approaches.

For example, in the field of biology and OMICS approaches, can the LinkML framework advantageously replace the ISA-Tools approach (Sanson et al., bioinformatics, 2012)? Or, concerning experiments involving a DoE (Design of Experiment) generating many data tables and having variables specific to each experiment, should we generate a new model each time? A comparison with the "Frictionless Data" framework (JSON format that could also integrate semantics, as in ODAM approach, Jacob et al. GigaScience, 2020) would be a plus.

There are certainly many frameworks and approaches that overlap to some extent with LinkML; however, we have not found any that completely cover all of LinkML's capabilities. In response to your suggestion, we have added a section in Conclusions about how LinkML compares with other relevant data modeling tools and frameworks. We have also added some additional text to the Conclusions section highlighting the features of LinkML that set it apart from related frameworks.

Sincerely,

Sierra Moxon (corresponding author)

Editorial Manager®

Cookie Preference Center

We use cookies which are necessary to make our site work. We may also use additional cookies to analyze, improve and personalize our content and your digital experience. For more information, see our Cookie Policy.

You may choose not to allow some types of cookies. However, blocking some types may impact your experience of our site and the services we are able to offer. See the different category headings below to find out more or change your settings.

Allow all

Manage Consent Preferences

Strictly Necessary Cookies

Always active

These cookies are necessary for the website to function and cannot be switched off in our systems. They are usually only set in response to actions made by you which amount to a request for services, such as setting your privacy preferences, logging in or filling in forms. You can set your browser to block or alert you about these cookies, but some parts of the site will not then work.

Functional Cookies

These cookies enable the website to provide enhanced functionality and personalisation. They may be set by us or by third party providers whose services we have added to our pages. If you do not allow these cookies then some or all of these services may not function properly.

Performance Cookies

These cookies allow us to count visits and traffic sources so we can measure and improve the performance of our site. They help us to know which pages are the most and least popular and see how visitors move around the site.

Targeting Cookies

These cookies may be set through our site by our advertising partners. They may be used by those companies to build a profile of your interests and show you relevant adverts on other sites. If you do not allow these cookies, you will experience less targeted advertising.

Cookie List

Clear

Apply Cancel

Consent Leg.Interest

Confirm my choices

Powered by onetrust
