# Supplementary material for: LinkML: an open data modeling framework
Source: Gigascience. 2025 Dec 12;15:giaf152. doi: 10.1093/gigascience/giaf152 (PMC12993438; doi:10.1093/gigascience/giaf152)
Supplement: Supplementary file 4 [file giaf152_reviewer_1_report_original_submission.pdf]

Reviewer Report

Title: LinkML: An Open Data Modeling Framework

Version: Original Submission **Date:** 7/28/2025

Reviewer name: Larry Lannom, MLS

Reviewer Comments to Author:

The assertion of the article is that the LinkML framework provides both high expressiveness and high ease of use. The authors do a good job of describing the framework, with a number of useful illustrations, and show its need in and application to biological microbiome sampling data. Those interested in such an approach will find the article to be a useful introduction.We have one recommended alteration. The authors assert that the approach complies with FAIR data standards, the first principle of which is persistent identification. While persistent identification is mentioned in numerous places in the article we don't find any detail on why the identifiers used are persistent. Perhaps readers more conversant in the domains from which the authors seem to come would understand this but we do not. We suggest a separate section or at least some detailed footnotes or citations to detail the way in which persistent identification fits within the LinkML framework.Co-reviewed with Robert Tupelo-Schneck (ORCID: https://orcid.org/0009-0008-8570-7342)

Level of Interest

Please indicate how interesting you found the manuscript: Choose an item.

Quality of Written English

Please indicate the quality of language in the manuscript: Choose an item.

Declaration of Competing Interests

Please complete a declaration of competing interests, considering the following questions:

- Have you in the past five years received reimbursements, fees, funding, or salary from an organisation that may in any way gain or lose financially from the publication of this manuscript, either now or in the future?
- Do you hold any stocks or shares in an organisation that may in any way gain or lose financially from the publication of this manuscript, either now or in the future?
- Do you hold or are you currently applying for any patents relating to the content of the manuscript?
- Have you received reimbursements, fees, funding, or salary from an organization that holds or has applied for patents relating to the content of the manuscript?
- Do you have any other financial competing interests?

- Do you have any non-financial competing interests in relation to this paper?

If you can answer no to all of the above, write 'I declare that I have no competing interests' below. If your reply is yes to any, please give details below.

I declare that I have no competing interests

I agree to the open peer review policy of the journal. I understand that my name will be included on my report to the authors and, if the manuscript is accepted for publication, my named report including any attachments I upload will be posted on the website along with the authors' responses. I agree for my report to be made available under an Open Access Creative Commons CC-BY license (http://creativecommons.org/licenses/by/4.0/). I understand that any comments which I do not wish to be included in my named report can be included as confidential comments to the editors, which will not be published.

Choose an item.

To further support our reviewers, we have joined with Publons, where you can gain additional credit to further highlight your hard work (see: https://publons.com/journal/530/gigascience). On publication of this paper, your review will be automatically added to Publons, you can then choose whether or not to claim your Publons credit. I understand this statement.

Yes Choose an item.
