# Supplementary material for: LinkML: an open data modeling framework
Source: Gigascience. 2025 Dec 12;15:giaf152. doi: 10.1093/gigascience/giaf152 (PMC12993438; doi:10.1093/gigascience/giaf152)
Supplement: Supplementary file 5 [file giaf152_reviewer_2_report_original_submission.pdf]

Reviewer Report

Title: LinkML: An Open Data Modeling Framework

Version: Original Submission **Date:** 8/4/2025

Reviewer name: Daniel Jacob

Reviewer Comments to Author:

The authors describe a very comprehensive open framework that allows various data to be described according to a structured metadata model. Undeniably, this framework seems to me to be of great use, both for the wealth of possibilities for describing complex data and for the associated tools for converting into different standard formats. The paper describes very well both the general approach and all the technical aspects of this framework, supported by examples.However, the authors have essentially limited themselves to a long technical description, which is extensively described in the online documentation. Although the article is of the "Technical Note" type, it seems to me that there is a critical section missing that positions the framework in the existing ecosystem, not only with regard to formats, but especially with regard to other approaches.For example, in the field of biology and OMICS approaches, can the LinkML framework advantageously replace the ISA-Tools approach (Sanson et al., bioinformatics, 2012)? Or, concerning experiments involving a DoE (Design of Experiment) generating many data tables and having variables specific to each experiment, should we generate a new model each time? A comparison with the "Frictionless Data" framework (JSON format that could also integrate semantics, as in ODAM approach, Jacob et al. GigaScience, 2020) would be a plus.The article seems well written to me, in very fluent English. However, as I am not a native English speaker, I will be careful not to correct the English.

Level of Interest

Please indicate how interesting you found the manuscript: Choose an item.

Quality of Written English

Please indicate the quality of language in the manuscript: Choose an item.

Declaration of Competing Interests

Please complete a declaration of competing interests, considering the following questions:

- Have you in the past five years received reimbursements, fees, funding, or salary from an organisation that may in any way gain or lose financially from the publication of this manuscript, either now or in the future?
- Do you hold any stocks or shares in an organisation that may in any way gain or lose financially from the publication of this manuscript, either now or in the future?

- Do you hold or are you currently applying for any patents relating to the content of the manuscript?
- Have you received reimbursements, fees, funding, or salary from an organization that holds or has applied for patents relating to the content of the manuscript?
- Do you have any other financial competing interests?
- Do you have any non-financial competing interests in relation to this paper?

If you can answer no to all of the above, write 'I declare that I have no competing interests' below. If your reply is yes to any, please give details below.

I declare that I have no competing interests

I agree to the open peer review policy of the journal. I understand that my name will be included on my report to the authors and, if the manuscript is accepted for publication, my named report including any attachments I upload will be posted on the website along with the authors' responses. I agree for my report to be made available under an Open Access Creative Commons CC-BY license (http://creativecommons.org/licenses/by/4.0/). I understand that any comments which I do not wish to be included in my named report can be included as confidential comments to the editors, which will not be published.

Choose an item.

To further support our reviewers, we have joined with Publons, where you can gain additional credit to further highlight your hard work (see: https://publons.com/journal/530/gigascience). On publication of this paper, your review will be automatically added to Publons, you can then choose whether or not to claim your Publons credit. I understand this statement.

Yes Choose an item.
